# Prostate-Specific Membrane Antigen–Targeted Imaging and Its Correlation with HOXB13 Expression

**DOI:** 10.2967/jnumed.123.267301

**Published:** 2024-08

**Authors:** Duminduni Hewa Angappulige, Nimrod S. Barashi, Nicholas Pickersgill, Cody Weimholt, Jingqin Luo, Ghazal Shadmani, Ziad Tarcha, Sampanna Rayamajhi, Nupam P. Mahajan, Gerald L. Andriole, Barry A. Siegel, Eric H. Kim, Kiran Mahajan

**Affiliations:** 1 Division of Urologic Surgery, Department of Surgery, Washington University in St. Louis, St. Louis, Missouri;; 2 Department of Pathology and Immunology, Washington University in St. Louis, St. Louis, Missouri;; 3 Division of Public Health, Department of Surgery, Washington University in St. Louis, St. Louis, Missouri;; 4 Alvin J. Siteman Cancer Center, Washington University in St. Louis, St. Louis, Missouri; and; 5 Division of Nuclear Medicine, Mallinckrodt Institute of Radiology, Washington University in St. Louis, St. Louis, Missouri

**Keywords:** prostate cancer, HOXB13, androgen receptor, PSMA, PSMA PET, metastasis

## Abstract

Homeobox 13 (HOXB13) is an oncogenic transcription factor that directly regulates expression of folate hydrolase 1, which encodes prostate-specific membrane antigen (PSMA). HOXB13 is expressed in primary and metastatic prostate cancers (PCs) and promotes androgen-independent PC growth. Since HOXB13 promotes resistance to androgen receptor (AR)–targeted therapies and regulates the expression of folate hydrolase 1, we investigated whether SUVs on PSMA PET would correlate with HOXB13 expression. **Methods:** We analyzed 2 independent PC patient cohorts who underwent PSMA PET/CT for initial staging or for biochemical recurrence. In the discovery cohort, we examined the relationship between HOXB13, PSMA, and AR messenger RNA (mRNA) expression in prostate biopsy specimens from 179 patients who underwent PSMA PET/CT with ^18^F-piflufolastat. In the validation cohort, we confirmed the relationship between HOXB13, PSMA, and AR by comparing protein expression in prostatectomy and lymph node (LN) sections from 19 patients enrolled in ^18^F-rhPSMA-7.3 PET clinical trials. Correlation and association analyses were also used to confirm the relationship between the markers, LN positivity, and PSMA PET SUVs. **Results:** We observed a significant correlation between PSMA and HOXB13 mRNA (*P* < 0.01). The association between HOXB13 and ^18^F-piflufolastat SUVs was also significant (SUV_max_, *P* = 0.0005; SUV_peak_, *P* = 0.0006). Likewise, the PSMA SUV_max_ was significantly associated with the expression of HOXB13 protein in the ^18^F-rhPSMA-7.3 PET cohort (*P* = 0.008). Treatment-naïve patients with LN metastases demonstrated elevated HOXB13 and PSMA levels in their tumors as well as higher PSMA tracer uptake and low AR expression. **Conclusion:** Our findings demonstrate that HOXB13 correlates with PSMA expression and PSMA PET SUVs at the mRNA and protein levels. Our study suggests that the PSMA PET findings may reflect oncogenic HOXB13 transcriptional activity in PC, thus potentially serving as an imaging biomarker for more aggressive disease.

## Visual Abstract

**Figure:**
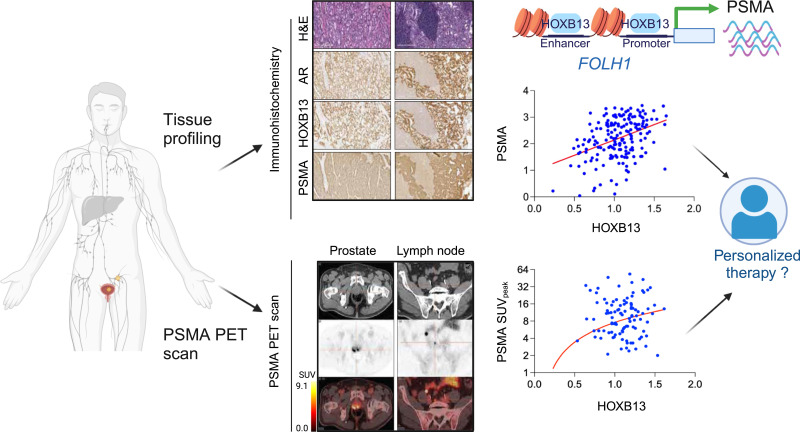

Biomarkers that can predict aggressive prostate cancers (PCs) early in development are critical to improve patient outcomes (1–4). One important biomarker of PC is prostate-specific membrane antigen (PSMA), which is overexpressed in most PCs (5,6); its higher expression is associated with castration-resistant disease (7,8) and inferior metastasis-free survival (9–11). Recently, the Society for Nuclear Medicine and Molecular Imaging and the National Comprehensive Cancer Network have recommended PSMA PET for the initial staging of patients with unfavorable intermediate-, high-, and very high–risk clinically localized PC, as well as for patients with biochemically recurrent PC (12,13). Thus, higher-risk patients may benefit from undergoing PSMA PET for treatment planning, with consideration of extended pelvic nodal dissection, pelvic nodal radiation, or the addition of chemohormonal agents (12).

PC demonstrates intra- and intertumor heterogeneity, which may increase in response to androgen deprivation (1,14). PSMA expression also demonstrates heterogeneity at the intra- and intertumoral levels (15–17). Although PSMA PET/CT has improved the detection of nonlocalized, recurrent, and metastatic PCs (18), understanding the molecular relationships associated with PSMA PET findings may enable improved selection of treatments to improve overall survival outcomes.

We and others have previously demonstrated that homeobox 13 (HOXB13) promotes androgen-independent growth of PC as a pioneer transcription factor and as a regulator of critical PC target genes including the androgen receptor (AR) and folate hydrolase 1 that encodes PSMA (3,13,19–23). Moreover, HOXB13 expression is increased in response to enzalutamide in PC cell lines, and its depletion increases enzalutamide sensitivity (3,22). As such, ours and other studies have reported the association of HOXB13 with more aggressive disease, specifically, in AR-negative castration-resistant PC (CRPC) and in some neuroendocrine PCs (3,21–26). To improve the understanding between clinical tools and molecular drivers of PC progression, we sought to evaluate the correlation between HOXB13, PSMA expression, and PSMA PET findings, as well as their association with lymph node (LN) metastasis.

## MATERIALS AND METHODS

### Study Design and Patient Demographics

**FIGURE 1.**
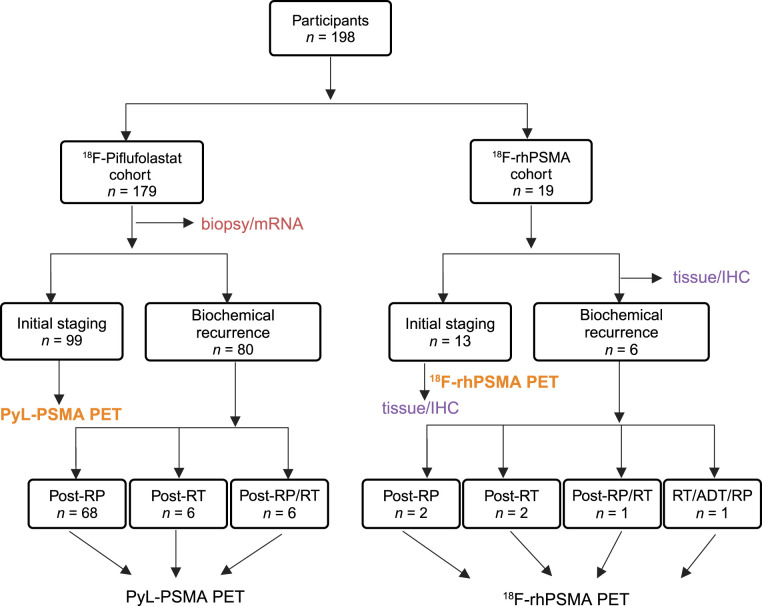
Study design of PSMA PET/CT and biomarker expression profiling in PC patients. Schematic diagram of patient stratification for retrospective analysis is shown. ADT = androgen-deprivation therapy; IHC = immunohistochemical; RP = radical prostatectomy; RT = radiation therapy.

The current study involving existing data was conducted under institutional review board approval with waiver of consent. We studied 2 independent subsets of patients imaged with 2 different PSMA radiopharmaceuticals. Group 1 was the discovery cohort, which included 179 patients who underwent PET/CT with ^18^F-piflufolastat (PyL) (Lantheus), and group 2 was the validation cohort, which included 19 patients who underwent PET/CT with ^18^F-rhPSMA-7.3 (Blue Earth Diagnostics) (Fig. 1). We performed a retrospective analysis of clinically obtained pathologic specimens (prostate biopsies before PSMA PET for group 1 and radical prostatectomy and LN sections after PSMA PET for group 2). Histopathologic examination was performed as a part of the standard clinical workflow for group 1 and group 2 specimens by board-certified genitourinary pathologists.

For the discovery cohort, messenger RNA (mRNA) expression profiling was performed using the Decipher GRID platform (Veracyte) (27). Normalized gene expression was obtained for all patients. For the validation cohort, deidentified formalin-fixed, paraffin-embedded (FFPE) radical prostatectomy and LN sections were used for immunohistochemical staining.

### Immunohistochemical Staining and Quantification

Two FFPE radical prostatectomy sections from each patient were selected for immunohistochemical staining and were sufficient for quantification. FFPE tissue sections were stained with hematoxylin and eosin and with specific antibodies for HOXB13, folate hydrolase 1, and AR. The hematoxylin and eosin–stained and immunohistochemical-stained slides were digitally scanned at ×20 magnification using an Aperio whole-slide scanner, and digital quantitation was performed with Aperio ImageScope software (Leica Biosystems). An Aperio Positive Pixel Count algorithm (version 9; Leica Biosystems) was used to perform the analysis (28). Average positivity of immunohistochemical staining was obtained as the ratio of the number of positive pixels divided by the total number of positive plus negative pixels. The immunohistochemical slides were also manually analyzed by a board-certified genitourinary pathologist using the following formula: quick score = *P* × *I*, where *P* represents the percentage of positive cells (0, 1+ [10%–25%], 2+ [25%–50%], 3+ [50%–75%], or 4+ [>75%]) and given as ordinal numbers and *I* represents intensity, scored as 1 (weak), 2 (moderate), or 3 (strong). More details are provided in the supplemental materials (supplemental materials are available at http://jnmt.snmjournals.org).

### PSMA PET Imaging and Quantification

Group 1 patients underwent PSMA PET/CT after intravenous injection of 333 MBq ± 20% of PyL. Imaging was performed with a Biograph 40HD or an mCT PET/CT scanner (Siemens Healthineers). Group 2 patients underwent PSMA PET/CT with 296 MBq ± 20% of ^18^F-rhPSMA-7.3 on a Siemens Biograph Vision 600 PET/CT scanner. The PSMA PET images were evaluated semiquantitatively by determination of SUV_max_ and SUV_peak_ of the most tracer-avid lesions identified on the scans (up to 3 foci in the prostate, if still present; up to 5 LN foci; and up to 2 osseous foci). In patients with multiple lesions in the prostate, the lesion with highest SUV_max_ was taken as the index lesion for correlation with mRNA or immunohistochemical analyses. For correlation of PSMA PET results with immunohistochemical results, the pathologist-defined lesion with the strongest (PSMA) or median (AR and HOXB13) pixel positivity was plotted against PSMA SUV_max_ or SUV_peak_. The PSMA PET and immunohistochemical parameters were correlated on a per-patient and per-lesion basis.

### Statistical Analysis

Unpaired *t* tests were used to compare mRNA and protein expression of genes between normal tissue and tumors. To compare patient and tumor characteristics between PET negative and PET positive and lymph node or bone metastasis versus no metastasis, Wilcoxon rank sum test was used for continuous characteristics, whereas a Fisher exact test was used for categoric ones. A linear regression model was fit on HOXB13 mRNA gene expression on PSMA PET results (based on SUV_max_ or SUV_peak_) and mRNA expression levels of AR, folate hydrolase 1, and prostate-specific antigen, adjusting for patient characteristics (age at scan, tumor stage, prostate-specific antigen level at scan, primary Gleason score, and time from radical prostatectomy). Pearson (parametric) and Spearman rank (nonparametric) correlation coefficients were calculated between 2 variables to quantify their correlation with *P* values reported testing the estimated correlation against 0. GraphaPad Prism (version 9.0) and R (version 4.4.0, http://cran.r-project.org) were used for data analyses. *P* values of less than 0.05 were considered statistically significant.

## RESULTS

### Clinicopathologic Outcomes and Patient Stratification by Treatment

A summary of the clinicopathologic variables of the PyL (group 1) and ^18^F-rhPSMA-7.3 (group 2) subjects is shown in Supplemental Tables 1 and 2. In group 1, of the 55.3% (99/179) treatment-naïve patients with newly diagnosed unfavorable intermediate-, high-, or very high–risk PC, according to the National Comprehensive Cancer Network, 93.9% (93/99) had positive PSMA PET results. Likewise, of the 44.6% (80/179) group 1 patients with biochemically recurrent PC, 47.5% (38/80) had positive PSMA PET results (Supplemental Table 1); group 2 consisted of 19 patients enrolled in the ^18^F-rhPSMA-7.3 clinical trials, for whom FFPE was available. PSMA PET revealed metastasis in 46% (6/13) of the treatment-naïve patients (2 bone and 4 LN metastases). In patients with biochemical recurrence, 66.6% (4/6) had PSMA PET–positive LNs (Supplemental Table 2). Pathologically positive LNs were found at radical prostatectomy or pelvic LN dissection in 46% (6/13) of treatment-naïve patients.

### Association of HOXB13 Expression with PSMA Tracer Uptake

**FIGURE 2.**
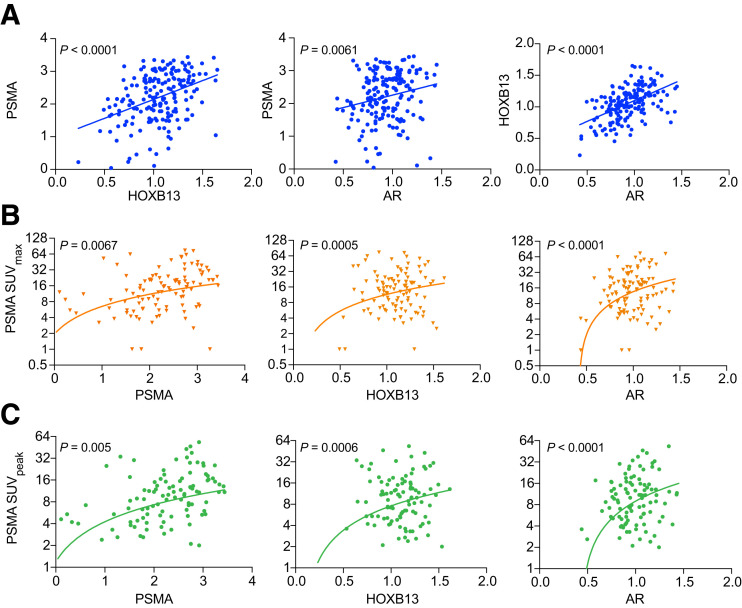
Association among HOXB13, PSMA, and AR with PSMA SUVs in PyL cohort. (A) Correlation among HOXB13, PSMA, and AR mRNA expression for each patient (*n* = 179). Lines represent linear regression. (B and C) Correlation among HOXB13, PSMA, and AR mRNA expression vs. prostate PSMA SUV_max_ (B) or prostate PSMA SUV_peak_ (C) for measurable lesions. Line represents linear regression (note logarithmic scale) (*n* = 105; initial staging PSMA PET–positive, *n* = 94; biochemical recurrence, *n* = 11).

Correlation analysis for PSMA, HOXB13, and AR mRNA expression in group 1 demonstrated strong correlation among each patient in this group (Fig. 2A). A significant correlation was also observed for the above 3 markers with the kallikrein genes *KLK2* and *KLK3,* encoding hexokinase 2 and prostate-specific antigen, respectively, according to the National Comprehensive Cancer Network guidelines on prostate-specific antigen serine proteases (Supplemental Figs. 1A–1C). In addition, PSMA and HOXB13 at mRNA levels (Spearman correlation [*r*] = 0.391; *P* < 0.0001) (Fig. 2A) and HOXB13 mRNA and PyL uptake showed significant correlations (*P* = 0.0005 and 0.0006 for SUV_max_ and SUV_peak_, respectively) (Figs. 2B and 2C). This correlation observation was also noted for AR (*P* < 0.0001 for SUV_max_ and SUV_peak_) and PSMA (*P* = 0.006 and 0.005 for SUV_max_ and SUV_peak_, respectively) (Figs. 2B and 2C). Linear regression analysis of HOXB13 against the covariates indicated association with the Gleason subpattern 5 versus 3 (*P* = 0.048), AR (*P* = 0.018), and PSMA (*P* = 0.022) mRNA expression (Supplemental Table 3).

### Coexpression of HOXB13 and PSMA in Primary PC and LN Metastases

**FIGURE 3.**
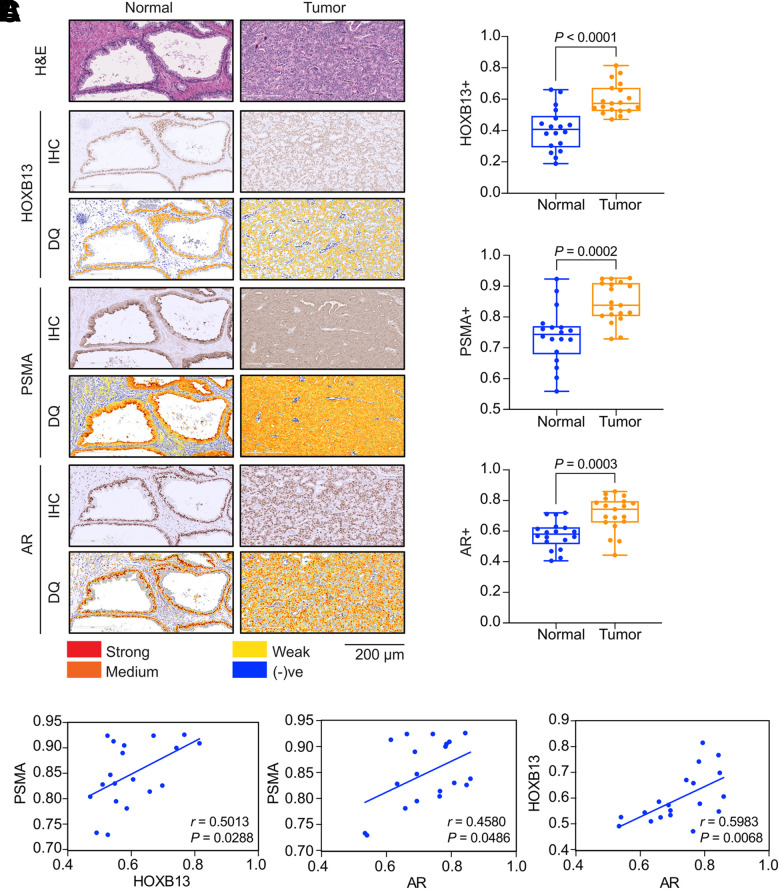
Correlation of HOXB13, PSMA, and AR protein expression in advanced PC. (A) Immunohistochemical (IHC) analysis shows representative normal and prostate tumor sections from individual FFPE patient specimens stained for AR, HOXB13, and PSMA. Digital quantification (DQ) analysis of marker expression is given below each panel. Scale bar = 200 μm. (B) Expression of HOXB13, AR, and PSMA in normal vs. tumor sections obtained by IHC and DQ determination was compared by Student *t* test (****P < 0.0001; *n* = 19). (C) Correlation analysis compares expression of each marker by IHC and DQ determination. Line represents linear regression, and *r* indicates Pearson correlation coefficient. H&E = hematoxylin and eosin; + = positive; (-)ve = negative.

PSMA, HOXB13, and AR protein expression was analyzed in group 2 FFPE specimens obtained from the prostate and LNs (Fig. 3A). Validation of the antibodies used for immunohistochemical analysis is provided in Supplemental Figures 2A and 2B. Digital quantification results of HOXB13, PSMA, and AR staining in these specimens are provided in Supplemental Table 4. All patient specimens had expression of the 3 targets with some degree of intra- and intertumoral heterogeneity (Supplemental Figs. 3A–3C). All 3 markers showed statistically significant differences between normal prostate and tumor, indicating increased protein expression overall in the tumor (Fig. 3B). Pearson correlation analysis comparing the expression of each marker in normal prostate and in tumor revealed a significant correlation between HOXB13 and PSMA for normal prostate (*r* = 0.684, *P* = 0.001) and tumor (*r* = 0.501, *P* = 0.028) (Fig. 3C; Supplemental Fig. 4A). This trend was maintained between AR and HOXB13, although slightly reduced in tumor (normal prostate: *r* = 0.804, *P* = <0.0001; tumor: *r* = 0.598, *P* = 0.006), and between PSMA and AR (normal prostate: *r* = 0.878, *P* < 0.0001; tumor: *r* = 0.458, *P* = 0.048) (Fig. 3C; Supplemental Figs. 4B and 4C).

**FIGURE 4.**
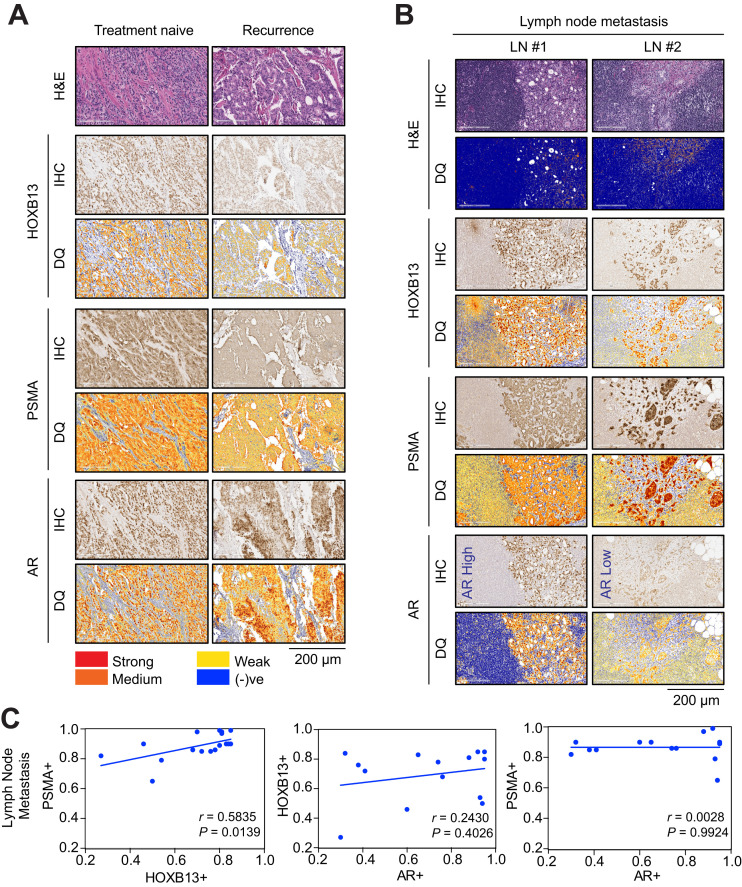
HOXB13 PSMA coexpression in recurrent and metastatic PC. (A) Representative normal and prostate tumor FFPE sections from treatment-naïve or recurrent PC immunohistochemically (IHC) stained for AR, HOXB13, and PSMA protein expression. Digital quantification (DQ) of staining is shown below each panel. Left panel: treatment-naïve. Right panel: recurrence with radiation plus androgen-deprivation therapy. (B) LN sections (GGG3 T3aN1 for LN 1 or GGG5 T3bN1 for LN 2). (C) Pearson correlation analysis comparing HOXB13 and PSMA protein expression in LNs. Line represents linear regression, and *r* indicates Pearson correlation coefficient. H&E = hematoxylin and eosin; + = positive; (-)ve = negative.

Among the patients in group 2 who had biochemical recurrence, sustained AR, HOXB13, and PSMA expression levels were identified despite prior radiation and androgen-deprivation therapy (Fig. 4A). Kruskal–Wallis testing that compared the treatment-naïve patients with the biochemical-recurrence patients revealed a significant difference for both AR (*P* < 0.00001) and HOXB13 (*P* < 0.00001) (Supplemental Fig. 5). Analysis of LN FFPE sections from treatment-naïve PC patients with PSMA PET–positive LNs revealed coexpression of HOXB13 and PSMA. However, AR expression was variable among these PSMA PET–positive LNs, with some displaying low levels of AR expression (Fig. 4B). Pearson correlation analysis revealed a significant correlation between HOXB13 and PSMA (*r* = 0.583, *P* = 0.014) but not between AR and HOXB13 (*r* = 0.243, *P* = 0.402) or PSMA and AR (*r* = 0.002, *P* = 0.992) in LN metastasis (Fig. 4C).

### HOXB13 and PSMA Expression and Correlation with PSMA SUVs

**FIGURE 5.**
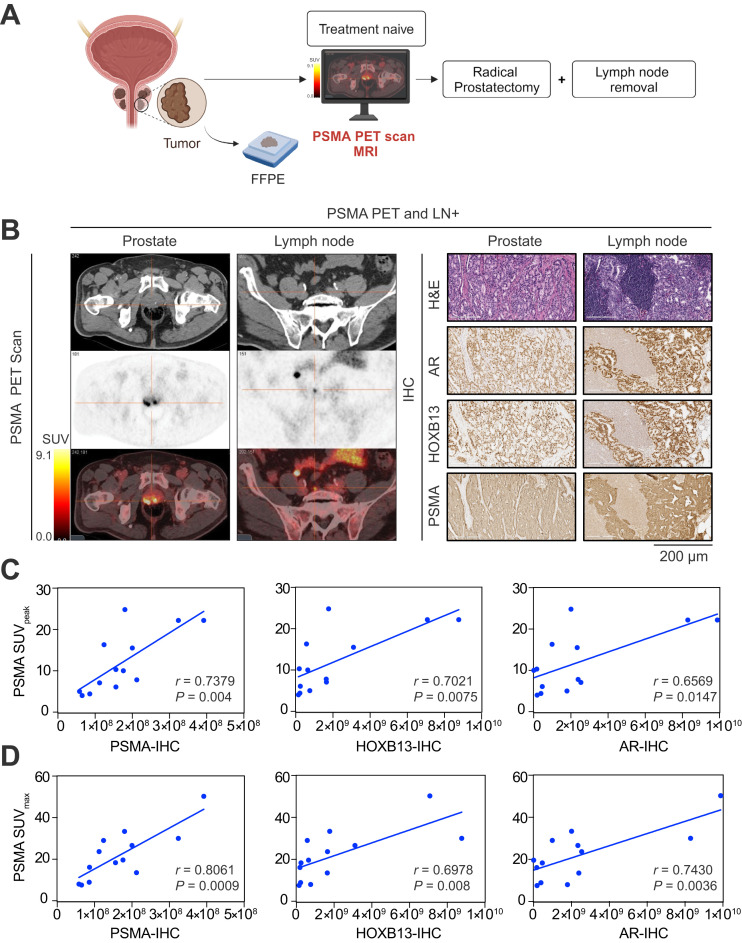
Detection of primary and metastatic prostate tumors by ^18^F-rhPSMA-7.3 PSMA PET. (A) Schematic of screening of PC patients with PSMA PET. (B) ^18^F-rhPSMA-7.3 PET/CT images of PSMA-positive prostate tumor and LN metastasis. Immunohistochemical (IHC) analysis shows representative FFPE-fixed prostate and LN patient sections stained for AR, HOXB13, and PSMA with corresponding hematoxylin and eosin (H&E) staining. Scale bar = 200 μm. (C and D) Pearson correlation analysis of the individual biomarker expression by IHC and digital quantification and prostate PSMA SUV_peak_ (C) (*n* = 19) and prostate PSMA SUV_max_ (D) (*n* = 19). Line represents linear regression, and *r* indicates Pearson correlation coefficient.

Analysis pipeline and corresponding immunohistochemical images of the PSMA PET scans for 2 representative cases are shown in Figures 5A and 5B. We observed statistically significant correlations for prostate tumor SUV_peak_ with immunohistochemical staining and digital quantitation for PSMA (*r* = 0.737, *P* = 0.004), HOXB13 (*r* = 0.702, *P* = 0.007), and AR (*r* = 0.659, *P* = 0.014) in the prostate lesions (Fig. 5C) or SUV_max_ with immunohistochemical staining and digital quantitation for PSMA (*r* = 0.806, *P* = 0.001), HOXB13 (*r* = 0.697, *P* = 0.008), and AR (*r* = 0.743, *P* = 0.003) in the prostate lesions (Fig. 5D; Supplemental Table 5).

## DISCUSSION

In this study, we found a significant relationship between HOXB13 and PSMA in PC. PC uptake of the PSMA tracers PyL and ^18^F-rhPSMA-7.3 was significantly associated with tissue-level HOXB13 expression, suggesting that PSMA PET findings may be an important prognostic biomarker for potentially lethal (i.e., castration-resistant) PC. An earlier study reported PSMA response heterogeneity in SUV_max_ in hormone-sensitive PC, whereas all men with metastatic CRPC showed an increase in SUV_max_ compared with that at baseline (17). In addition, for patients with advanced PC started on abiraterone and enzalutamide, a change in PyL uptake in PC lesions or development of new lesions was prognostic of time-to-therapy change and overall survival (29). This difference in PSMA response may be partially attributable to heterogeneous HOXB13 and AR expression as their levels significantly change in CRPCs compared with hormone-naïve PCs (26,30).

Our study findings are consistent with prior publications that have demonstrated PSMA expression positively correlating with higher Gleason scores (*P* < 0.0001 in biopsy specimens and *P* = 0.007 in prostatectomy samples) (31), with LN involvement (*P* = 0.007) (32) and reduced recurrence-free survival (*P* < 0.001) (33). In a treatment scenario, hormone-naïve men treated with androgen-deprivation therapy showed a reduction in PSMA tracer uptake initially on treatment, with a subsequent rise at some tumor sites (17,34). Furthermore, PSMA levels and ^68^Ga-PSMA-11 uptake are increased in men with metastatic CRPC treated with enzalutamide (7,17,35,36). Thus, the clinical importance of PSMA PET imaging lies in its ability to redefine staging via its sensitivity to detect metastatic disease in patients with previously diagnosed nonmetastatic CRPC (37). Most importantly, the future of PC treatment will likely include ^177^Lu-PSMA therapy, as it has demonstrated high response rates, low toxicity, and improved quality of life for men diagnosed with metastatic CRPC, despite prior treatment with docetaxel, cabazitaxel, or a second AR pathway antagonist, abiraterone, after enzalutamide (38,39).

This relatively novel description of the strong relationship between expression of HOXB13 and PSMA with the results of PSMA PET has several potential clinical implications. For example, HOXB13 expression in biopsy specimens might be useful to guide the appropriateness of PSMA PET for initial staging. Currently, the National Comprehensive Cancer Network’s risk categories are used to determine who may benefit from PSMA PET, which is not personalized to an individual patient’s PC biology. With HOXB13 expression and activity linked to castration-independent behavior (21,22,24,25,40), patients with high HOXB13 and PSMA expression may benefit from primary treatments that do not rely on androgen-deprivation therapy or castration to be therapeutically effective. Furthermore, HOXB13 and PSMA expression may be useful as a biomarker to better select patients for earlier use of PSMA-targeted radiopharmaceutical therapy, such as ^177^Lu-PSMA-617 (41–43). In the currently ongoing trial PSMAddition (NCT04720157), which is comparing ^177^Lu-PSMA-617 therapy plus the standard of care with the standard of care alone in newly diagnosed metastatic hormone-sensitive PC, secondary analysis of diagnostic prostate biopsy tissue for HOXB13 expression may reveal which patients are better suited for upfront PSMA-targeted radiopharmaceutical therapy.

The use of different PSMA tracers could be considered a limitation of our study. However, this is reflective of clinical practice, and in this study, the correlation with HOBX13 was maintained, irrespective of the specific tracer used. The LN studies are underpowered, and thus the significance of the finding remains to be determined. Technical limitations may have impacted colocalization between FFPE tissue samples and PET activity. However, the significant positive correlation we observed between markers in pathologist-confirmed lesions and PSMA PET findings in our large cohort suggests that we were able to overcome this limitation.

The correlations between HOXB13 and PSMA expression are statistically significant, but the magnitude of correlation is limited in the discovery cohort (group 1). This limited correlation may be due to gene expression methodology performed with Decipher GRID (Decipher Bioscience). Previously, we reported a strong correlation between PSMA and HOXB13 expression in The Cancer Genome Atlas Prostate Adenocarcinoma (*n* = 492, *r* = 0.51, *P* = 2.2 × 10^–37^) and the Stand Up to Cancer–Prostate Cancer Foundation (*n* = 444, *r* = 0.54, *P* = 3.66 × 10^–10^) datasets, which are based on RNA sequencing (3). Compared with RNA sequencing, the Decipher GRID microarray hybridization technology may have limited gene-expression measurements because of the background signal at the low end and signal saturation at the high end (>10^5^ for RNA sequence vs. 10^3^ for arrays).

## CONCLUSION

HOXB13 expression at both mRNA and protein levels correlates significantly with clinical PSMA PET findings, underscoring the likely mechanistic relationship between HOXB13 and PSMA expression in PC. Our findings of PSMA PET association with HOXB13 could pave the way for future studies incorporating HOXB13 and PSMA testing at earlier stages of PC diagnosis, which could then guide personalized PC care.

## DISCLOSURE

Kiran Mahajan received financial support from the Department of Defense (W81XWH-21-1-0203) and the Department of Surgery at Washington University. Eric Kim is supported by funding from the Midwest Stone Institute, the American Cancer Society, the Siteman Investment Program, and NIH/NCI grant R01-CA258690. PET/CT analysis was supported in part by NCI Cancer Center support (P30 CA091842). Barry Siegel reports receiving grants from the American College of Radiology, Blue Earth Diagnostics, Curium Pharma, and Progenics Pharmaceuticals and receiving personal fees from the American College of Radiology, Capella Imaging, Curium Pharma, Evicore Health Care, GE Healthcare, Lantheus Medical Imaging, and Siemens Healthineers. Gerald Andriole has an advisory role with Invitae, Lantheus Medical Imaging, and Siemens Medical Solutions. Kiran Mahajan and Nupam Mahajan are cofounders of Technogenesys, a startup company that controls the intellectual property and patents on the ACK1 inhibitor (*R*)-**9b**. No other potential conflict of interest relevant to this article was reported.

## Notes

KEY POINTS **QUESTION:** Does the uptake of PSMA-targeted radiopharmaceuticals correlate with HOXB13 expression in PC lesions to enable improved molecular profiling of PC? **PERTINENT FINDINGS:** Prostate tumor SUVs of PyL and ^18^F-rhPSMA-7.3 were significantly associated with HOXB13 expression as assessed by mRNA analysis or by immunohistochemical staining. **IMPLICATIONS FOR PATIENT CARE:** Combining PSMA PET with HOXB13 RNA or protein expression profiling of biopsy specimens or resected tumors may enable early improved profiling of high-risk PC in the clinical setting.
